# The Association Between Hyperkalemia, Necrotic Bowel, and Cardiac Arrest Episodes: A Case Report

**DOI:** 10.7759/cureus.66490

**Published:** 2024-08-09

**Authors:** Rubin Xu, Chunyan Wang, Wenli Yu, Eva Zhang, Muzi Meng

**Affiliations:** 1 Anesthesiology, Tianjin First Central Hospital, Tianjin, CHN; 2 Anesthesiology, Tianjin Medical University General Hospital, Tianjin, CHN; 3 Surgery, Mount Sinai Hospital, New York, USA; 4 Surgery, St. George's University, True Blue, GRD; 5 School of Medicine, American University of the Caribbean, Cupecoy, SXM; 6 General Surgery, BronxCare Health System, The Bronx, USA

**Keywords:** cardiac resuscitation, cardiac arrest, bowel ischemia, bowel necrosis, hyperkalemia

## Abstract

This case report presented the details of the case and outcomes of the relevant literature review to offer insights into how hyperkalemia triggers cardiac arrest and the resuscitation rate of patients with hyperkalemia. This report discusses how conditions such as hyperkalemia and necrotic bowel can lower patients' heart rate, with regard to patients' baseline cardiac condition. This report also justifies the possibility of patients experiencing cardiac arrest episodes before and after chest/abdomen surgeries.

## Introduction

Hyperkalemia is a potentially life-threatening condition characterized by elevated potassium levels in the blood, which can weaken cardiac muscles and significantly increase the risk of cardiac arrhythmias and cardiac arrest [1]. The high potassium levels disrupt the normal electrical activity of the heart, making it more susceptible to irregular rhythms that can be fatal if not promptly managed [1]. This condition can also contribute to the development of bowel necrosis, a severe and potentially fatal complication where parts of the bowel tissue die due to lack of blood flow [2]. Bowel necrosis may further lead to a strangulated hernia, a critical situation where the blood supply to the herniated tissue is cut off, causing severe pain and potentially life-threatening complications if not treated swiftly.

Patients with hyperkalemia are particularly vulnerable to cardiac complications due to the heightened sensitivity of their cardiac muscles to compressions and pressures [3]. This sensitivity is especially pronounced during medical interventions such as cardiopulmonary resuscitation (CPR) or cardio-related surgical procedures, where the mechanical stress on the heart can exacerbate the risk of arrhythmias [3].

In the case presented in this report, the patient experienced multiple episodes of cardiac arrest associated with severe hyperkalemia and necrotic bowel. Notably, these cardiac arrest episodes occurred both before and after surgical resection of the necrotic intestine. This correlation underscores the complex interplay between hyperkalemia and the increased risk of cardiac arrest in patients with compromised bowel and cardiac health.

## Case presentation

The patient is a 69-year-old male with a past medical history of diabetes, cerebral infarction, and right inguinal hernia for 10 years, who presented to the ED with a one-hour history of lower-right inguinal pain without nausea or vomiting. The patient was in generalized discomfort. His temperature was normal, his blood pressure was 170/95 mmHg, and his heart rate was 81 beats per minute (bpm). On examination, there was a firm, non-reducible right inguinal hernia, measuring around 10×10 cm, tender to palpation.

**Figure 1:**
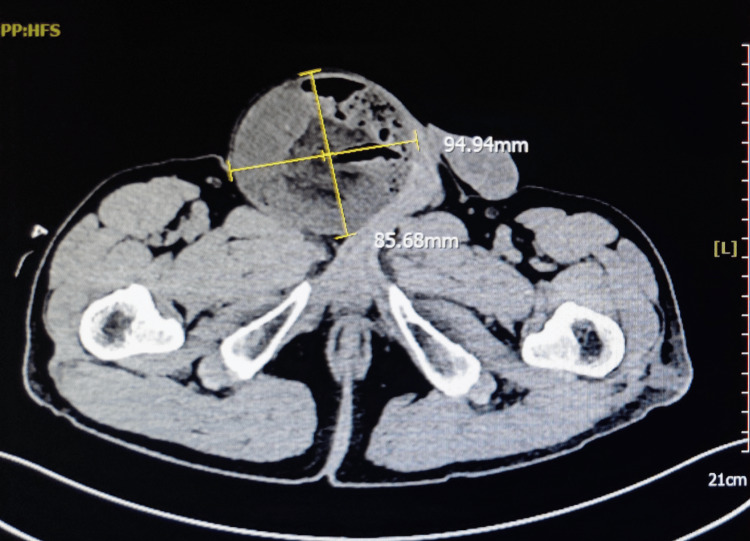
CT of the abdomen and pelvis CT of the abdomen and pelvis without contrast revealed a right-sided inguinal hernia with multiple loops of small bowel within the sac, with the largest cross-sectional area being approximately 9.4×8.8 cm

CT of the abdomen and pelvis without contrast revealed a right-sided inguinal hernia with multiple loops of small bowel within the sac, with the largest cross-sectional area being approximately 9.4×8.8 cm (Figure 1). There was also significant fat-stranding of the adjacent soft tissue.

The patient was brought to the OR for emergent exploratory laparotomy due to concerns of a strangulated inguinal hernia with possible bowel necrosis. At the beginning of the surgery, the laboratory values were noted (Table 1). General anesthesia was performed. Anesthesia was induced with midazolam 2 mg, sufentanil 30 µg, etomidate 16 mg, and rocuronium 60 mg. For maintenance of anesthesia, propofol was administered at a rate of 200-400 mg/h and remifentanil at 0.3-1 mg/h, and rocuronium was given as a bolus of 40 mg every hour.

**Table 1:** Laboratory findings WBC: white blood cell; CRP: C-reactive protein

Parameter	Value (initial)	Value (pre-surgery)	Value (intraoperative)	Value (cardiac arrest)	Value (post-resuscitation)	Reference range
WBC count (×10^9^/L)	9.94	-	-		-	4.0-11.0
CRP (mg/L)	1.25	-	-		-	<5.0
Serum sodium (mEq/L)	143	140	137	148	141	135-145
Serum potassium (mEq/L)	3.26	3.60	5.9	6.1	3.7	3.5-5.0
Arterial lactate (mmol/L)	2.9	2.9	1.7	4.6	8.3	0.5-2.2

An oblique incision was made above the right inguinal ligament, and the hernia sac wall was cut open, revealing the necrotic small intestine as the hernia content. Due to difficult exposure, the herniated content was reduced, and the incision was extended. Severe adhesions were found in the abdominal cavity, which were then lysed. Careful exploration of the abdominal cavity revealed approximately 50 ml of dark red exudate. At about 120 cm from the ileocecal junction, a necrotic segment of the ileum, about 120 cm long, was observed. The small bowel mesentery was sequentially clamped and divided, and the bowel was clamped at the distal and proximal ends of the necrotic segment and then divided, and the specimen was completely removed (opening the necrotic bowel revealed a large amount of chymous and bloody intestinal fluid inside). After disinfection, a side-to-side anastomosis of the small bowel was performed using a stapler. 

About 5-10 minutes after the resection of the necrotic ileum and side-to-side anastomosis of the small intestine, supraventricular tachycardia occurred. Lidocaine 100 mg was administered, and bedside blood gas analysis was performed simultaneously. The heart rate dropped to around 90 bpm, and the blood gas analysis results showed hyperkalemia (Table 1). The heart rate quickly decreased to asystole (approximately 15 seconds). Before complete cardiac arrest, the surgeon immediately performed chest compressions and sequentially administered intravenous epinephrine 1 mg, sodium bicarbonate 150 ml, insulin 8 IU, lidocaine 100 mg, defibrillation at 360 J, epinephrine 1 mg, calcium chloride 1 g, epinephrine 1 mg, defibrillation at 360 J, epinephrine 1 mg, and insulin 8 IU. About 10 minutes later, the heartbeat resumed. After resuscitation, an additional 8 IU of insulin and 100 ml of sodium bicarbonate were administered based on the blood gas results (Table 1).

The patient was not extubated and shifted to the ICU for further management in the postoperative period. He remained hemodynamically stable and was extubated on postoperative day 6, and, following an uneventful hospital course, the drain was removed on postoperative day 16, and the patient was discharged on postoperative day 16. Upon discharge, the patient remained afebrile with a temperature of 36.8℃, with a downtrended white blood cell (WBC) level of 13.61×10^9^/L. The patient followed up with the outpatient clinic with no complications to date.

The resected small bowel was sent to pathology, which showed "necrosis and bleeding of the small intestine, with dilated and congested intestinal blood vessels; reactive hyperplasia of the mesenteric lymph nodes with bleeding."

## Discussion

Hyperkalemia and cardiac arrest

Hyperkalemia is a condition where a patient's potassium level exceeds 5.0 mEq/L, with emergent hyperkalemic episodes occurring when potassium levels rise above 6.5 mEq/L [1]. Elevated potassium levels pose significant risks because potassium is crucial for cellular function, and any substantial change can lead to dysfunction in muscles, nerves, and the heart [1]. The relationship between fluctuating potassium levels and the risk of arrhythmias, conditions characterized by abnormal heart rhythms, is well-documented, with the risk of arrhythmias increasing in tandem with rising potassium levels. If hyperkalemia is not promptly managed, it can escalate to fatal cardiac arrhythmias [1].

Patients with chronic kidney disease are at a higher risk for hyperkalemia, but other contributing factors include hypertension, congestive heart failure, diabetes mellitus, liver disease, and coronary artery disease [4]. Diabetes mellitus can exacerbate the risk of hyperkalemia through multiple mechanisms. Insulin plays a crucial role in driving potassium into cells [5]. In patients with diabetes, especially those with poor glycemic control or insulin deficiency, this mechanism is impaired, leading to higher levels of potassium in the blood. Furthermore, diabetic nephropathy, a common complication of long-standing diabetes, can impair renal function and decrease the kidney's ability to excrete potassium. Additionally, certain medications commonly used in diabetes management, such as angiotensin-converting enzyme (ACE) inhibitors or angiotensin II receptor blockers (ARBs), can further inhibit potassium excretion, compounding the risk [5]. Therefore, it is essential to monitor and manage potassium levels meticulously in diabetic patients to prevent hyperkalemia and its associated complications.

Interestingly, tissue necrosis may also precipitate hyperkalemia, implying that necrotic bowel conditions can trigger hyperkalemia and subsequently lead to cardiac arrest. Hyperkalemia reduces the resting membrane potential in both striated and smooth muscle, increasing cardiac depolarization and muscle excitability [4]. This heightened excitability can precipitate various life-threatening conditions, including sinus wave patterns, ventricular fibrillation, idioventricular rhythms, and, ultimately, cardiac arrest [4]. The insidious nature of hyperkalemia lies in its often asymptomatic presentation. Most patients with elevated potassium levels do not exhibit overt symptoms, making it imperative to detect changes through routine screening labs [6]. Early detection and management are critical to preventing the severe cardiovascular complications associated with hyperkalemia.

Intestinal necrosis and cardiac arrest

Intestinal necrosis occurs when the gastrointestinal tract receives insufficient blood flow, leading to the death of intestinal cells [7]. This condition, also known as intestinal ischemia, is a severe and potentially life-threatening condition [6]. In adults, the primary causes of intestinal necrosis include acute mesenteric occlusion, chronic ischemia, and inflammatory diseases. Necrosis is often a late-stage manifestation of significantly reduced blood flow to the gastrointestinal tract.

We hypothesized that the necrotic material from the ischemic necrotic bowel entering the bloodstream would cause hyperkalemia. Reperfusion of the ischemic necrotic bowel leads to the disruption of electrolyte balance and the internal environment [2]. The patient had hypokalemia prior to the start of surgery and experienced an abrupt increase in potassium during surgery. During the procedure, the incarcerated hernia was reduced after the localization of the ischemic bowel. Since there was poor restoration of blood flow, bowel resection was decided. The patient was hypotensive at the time of bowel resection and had supraventricular tachycardia and cardiac arrest soon after resection. It is hypothesized that intestinal ischemia-reperfusion has occurred and the reperfusion injury led to intestinal necrotic substances being released into the bloodstream. The release of such substances ultimately is responsible for the disruption of homeostasis and the internal environment.

According to Hoftun Farbu et al., there is a strong association between intestinal ischemia and prolonged episodes of cardiac arrest, suggesting that patients with bowel necrosis are at a high risk of experiencing heart stoppages [8]. The progression from intestinal necrosis to cardiac arrest can be attributed to the severe systemic effects of ischemia, which include end-organ damage, hepatic failure, and hypotension [6]. Even if patients survive initial interventions, they remain at significant risk for developing comorbid conditions and, in some cases, long-term disabilities [7].

Alyonan further elucidates that several factors can cause intestinal ischemia, such as poor cardiac output, venous drainage blockage, embolism, and thrombosis [9]. These factors can act in isolation or combination, compounding the severity of the condition. Additionally, the study highlights that intestinal necrosis can result from cardiac arrest, indicating a bidirectional relationship where each condition can precipitate the other. This relationship underscores the complexity of managing patients with compromised cardiovascular and gastrointestinal systems [9]. 

Moreover, patients with extensive colonic necrosis may still experience cardiac arrest despite successful CPR, illustrating the limitations of CPR in such scenarios [10]. This observation implies that conventional resuscitation techniques might not be sufficient for patients with severe bowel necrosis, necessitating more advanced and targeted therapeutic strategies.

Given that both bowel necrosis and heart failure are circulatory diseases, it is not far-fetched to establish a link between the two. The interplay between reduced blood flow and systemic ischemia highlights the critical need for comprehensive management of both gastrointestinal and cardiac conditions to improve patient outcomes. 

CPR and rescue for cardiac arrest

CPR is a critical set of life-saving interventions designed to provide oxygenation and circulation to the body during cardiac arrest [11]. Properly performed CPR can supply up to 33% of normal cardiac output and oxygenation, making it a crucial first-aid measure in urgent situations like cardiac arrest [11]. The timely application of CPR is essential; it should be initiated when an unresponsive patient has no detectable pulse. Prompt action is vital because the sooner CPR is started after the heart stops pumping blood and oxygen to the brain and other organs, the higher the chances of survival without severe brain damage [3].

In patients with conditions such as strangulated hernia and necrotic bowel, the urgency of CPR becomes even more pronounced. These conditions can lead to severe complications, including necrosis of the abdominal wall hernia and ischemia of a portion of the distal sigmoid colon, which may precipitate cardiac arrest [12]. The mechanical pressure from a hernia can compromise circulatory function, escalating to cardiac arrest if not promptly addressed [13].

Intriguingly, the application of CPR itself carries certain risks, particularly in patients with preexisting conditions [14]. High-quality chest compressions, while essential for effective CPR, can sometimes cause severe thoracic injuries. For instance, lung herniation has been documented as a result of vigorous chest compressions during CPR. The use of mechanical chest compression devices can increase the risk of such injuries, highlighting the need for careful monitoring during resuscitation efforts [14]. Chest X-rays often reveal rib fractures and loculated air pockets, which can create conditions conducive to hernia formation. Notably, hernias may develop even in the absence of rib fractures, complicating the clinical picture [14].

CPR and resuscitation success

The incidence of cardiac arrest following major cardiac surgery ranges from 0.7% to 2.9%, indicating that this condition, while relatively uncommon, is a significant concern [15]. Immediate and effective intervention is critical to ensure successful resuscitation. External chest compressions, a key component of CPR, can sometimes lead to cardiac damage, especially in the context of repeated defibrillation attempts. Cardiac arrest management often involves up to three successive defibrillation attempts, which can increase the risk of cardiac injury [15].

There is a notable discrepancy between public perception and the actual success rates of CPR. Research indicates that both clinicians and the general public tend to overestimate the success rates of CPR, with many believing that the success rate exceeds 75% in all situations [16]. This overestimation highlights a lack of public understanding about the factors that influence CPR outcomes, such as the patient's underlying health conditions.

Patients with hyperkalemia present a particular challenge for resuscitation. Studies by Kose and Bilgin have shown that CPR may often fail to resuscitate patients with hyperkalemia. In such cases, hemodialysis may be necessary as a follow-up treatment to manage hyperkalemia and support resuscitation efforts [4]. This suggests that standard CPR may need to be supplemented with additional medical interventions to effectively resuscitate patients experiencing cardiac arrest due to hyperkalemia. Furthermore, the application of CPR can lead to complications such as strangulated hernia, especially in patients with underlying conditions like intestinal necrosis [10]. Intense chest compressions can exacerbate preexisting conditions, leading to further medical complications. The risk of intestinal necrosis and subsequent hernia development underscores the need for careful assessment and management of patients undergoing CPR.

## Conclusions

Hyperkalemia is a critical condition that can precipitate cardiac arrest due to the abrupt elevation of potassium levels, which weakens cardiac muscles and disrupts normal heart function. This case report highlights a complex medical scenario where a strangulated hernia and resultant bowel necrosis led to hyperkalemia, ultimately causing cardiac arrest. It demonstrates how a strangulated hernia can progress to bowel necrosis due to insufficient blood flow, leading to significant cellular death within the bowel. This necrosis, in turn, releases high levels of potassium into the bloodstream, resulting in hyperkalemia. Elevated potassium levels disrupt normal cardiac function, increasing the risk of severe arrhythmias and cardiac arrest. The presence of necrotic bowel tissue complicates the clinical picture and poses additional risks during resuscitation efforts, as aggressive CPR can exacerbate these conditions.

The findings emphasize the complexity of managing patients with concurrent hyperkalemia and gastrointestinal pathology. The resuscitation rate in such patients is lower than commonly perceived, necessitating a tailored approach that considers the interplay of these conditions. This case underscores the importance of timely diagnosis and comprehensive treatment strategies to mitigate the risks associated with hyperkalemia and its complications. Early detection and management of hyperkalemia are critical to preventing severe cardiovascular complications, particularly in patients with diabetes, who are at an increased risk due to impaired potassium regulation and potential nephropathy.
